# Real-time detection of antibiotics cytotoxicity in rabbit periosteal cells using microfluidic devices with comparison to conventional culture assays

**DOI:** 10.1186/s12891-019-2705-y

**Published:** 2019-07-26

**Authors:** Chih-Hao Chiu, Kin Fong Lei, Yi-Sheng Chan, Steve W. N. Ueng, Alvin Chao-Yu Chen

**Affiliations:** 1 Bone and Joint Research Center, Department of Orthopedic Surgery, Chang Gung Memorial Hospital-Linkou and University College of Medicine, 5th, Fu-Shin Street, Kweishan Dist, Taoyuan, 333 Taiwan, Republic of China; 2 Graduate Institute of Medical Mechatronics, Chang Gung University, Taiwan, Republic of China

**Keywords:** Impedance, Periosteum, Antibiotics, Cytotoxicity, Microfluidic, Cell index, WST-1, Osteogenic

## Abstract

**Background:**

Local antibiotic application has been widely used in orthopedic surgery. The dose-related toxicity of antibiotics towards periosteal tissues and resulting effects on osteogenic expression are yet to be studied.

**Methods:**

Periosteal cells harvested from the medial tibia of New Zealand White rabbits were used. A seeding density of 5 × 10^3^ cells/cm^2^ was determined to be optimal for testing in the pilot study; the cells were cultured in xCELLigence 96-well plates. Microfluidic impedance analyzers were used to monitor cellular proliferation in microfluidic culture systems with exposure to three different concentrations (10 μg/mL, 100 μg/mL, and 1000 μg/mL) of cefazolin, ciprofloxacin, and vancomycin, respectively. The correlation of cell index at day 7 with optical density values from WST-1 assays using conventional cultures was evaluated by calculating the Pearson’s coefficient. RNA analysis was performed to investigate the expression of osteogenic markers in the cultured cells, including core-binding factor alpha 1 (Cbfa1), osteopontin (OPN), and osteopontin promoter (OPNp), relative to glyceraldehyde-3-phosphate dehydrogenase (GAPDH) as the endogenous control.

**Results:**

A significant dose-related inhibition of cell index was found for all the 3 antibiotics, whereas the WST-1 assays showed a significant dose-related inhibition of cellular proliferation only at a high dose of cefazolin (1000 μg/mL) and medium-to-high dose of ciprofloxacin (100 μg/mL and 1000 μg/mL). Pearson’s coefficient analysis indicated a high correlation between the cell index and optical density values of WST-1 assays only for medium and high doses of ciprofloxacin (100 μg/mL and 1000 μg/mL); a moderate correlation was seen for cefazolin, and a low dose of ciprofloxacin (10 μg/mL). RNA analysis confirmed significant dose-related inhibition of cfba1, OPN, and OPNp expression by all three antibiotics.

**Conclusion:**

With optimal seeding amounts, rabbit periosteal cells can be dynamically monitored in the xCELLigence microfluidic system. Dose-related inhibition of cellular proliferation and osteogenic expression was found after exposure to cefazolin and ciprofloxacin. By providing real-time detection and exhibiting comparable correlation, microfluidic impedance-based analyzer is a feasible alternative to the conventional WST-1 assays.

## Background

Local injections often are used in the management of common musculoskeletal conditions [1–3]. Among these agents, antibiotics have been empirically used for the prevention and treatment of bone and joint infection for several decades [4]. Antibiotic-loaded carriers were designed not only to eradicate bone infection but also to facilitate trabecular bone formation [5]. However, currently, there are no available data regarding the dose-related cytotoxicity of antibiotics towards periosteal tissues, and their resulting effects in osteogenesis have not yet been meticulously surveyed.

A commercial microfluidic cell analyzer called the xCELLigence system (xCELLigence, Roche/ACEA Biosciences, CA) performs an impedance-based analysis, allowing the label-free dynamic monitoring of relatively viable and adherent cell amounts [6] and has been applied for the real-time detection of cell migration and proliferation in cancer immunotherapy [7], cytotoxicity [8], and drug resistance research [9]. Recently, this technology has been exploited to investigate the cellular profiles of osteoprogenitors derived from human jaw periosteum; it has shown potential to be used for engineering applications in maxillofacial orthopedic surgery [10].

In this study, we proposed a non-invasive, label-free research model in cultured cells. Rabbit periosteal cell proliferation and responses to different drugs were recorded using both the xCELLigence system and conventional cell proliferation assays. End-point results of cell proliferation were compared by using the two methods. We hypothesize that the xCELLigence biosensor technology can serve as a valuable platform for the real-time monitoring of rabbit periosteal cell behavior and responses to different stimuli.

## Methods

We used animals from the Laboratory Animal Center, Chang Gung Memorial Hospital-Linkou. Written informed consent (2015122504) was obtained from the Institutional Animal Care and Use Committee (IACUC) to use the animals for executing this study. The animal use protocol listed below has been reviewed and approved by the IACUC, and the Committee recognized that the proposed animal experiment follows the Animal Protection Law by the Council of Agriculture, Executive Yuan, R.O.C. and the guideline as shown in the Guide for the Care and Use of Laboratory Animals s promulgated by the Institute of Laboratory Animal Resources, National Research Council, U.S.A. After completion of the study, all the experimental animals were taken care by the Laboratory Animal Center and ultimately euthanized with intramuscular Zoletil 50 (0.5 mL/kg) and intravenous 2% Lidocaine (5 mL) on reaching humane endpoints.

### Periosteum harvest and isolation of cells

**Fig. 1:**
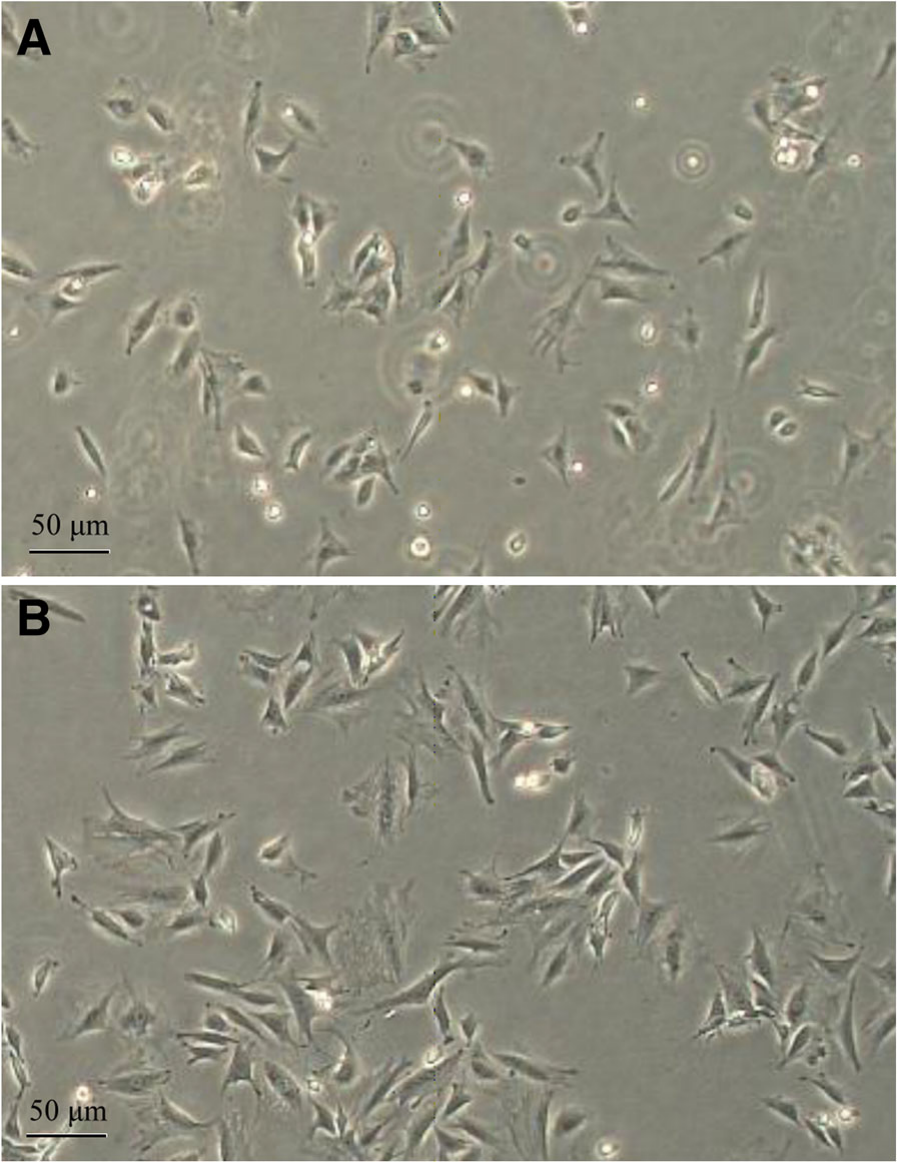
Microscopic morphology of periosteal cell culture. **a** Second passage. **b** Third passage

Under anesthesia by using intramuscular injection of Zoletil 50 (0.5 mL/kg) and sterile conditions, a 3-cm skin incision was made along the medial tibias of 6 adult male New Zealand White rabbits in a single animal group. The harvested periosteum was sectioned into pieces washed twice in calcium- and magnesium-free Dulbecco’s phosphate-buffered saline (DPBS) and then finely minced. The cells were released by treatment with 0.3% collagenase II for 2 h in low-glucose Dulbecco’s modified Eagle’s medium (LG DMEM) containing antibiotic solution (100 U/mL penicillin and 100 mg/mL streptomycin) with gentle agitation. After the same volume of DPBS was added, the undigested tissue was removed using a 100-mm nylon sieve. After centrifugation, cells were collected and then washed twice. Finally, for further use, they were re-suspended in LG DMEM supplemented with 10% fetal bovine serum (FBS) and antibiotic solution. The genotype of the periosteal cells was confirmed by real-time polymerase chain reaction (PCR) for detecting the osteogenic markers. Normal morphologic characteristics were confirmed by microscopy with elongated change of periosteal cells noted in differentiating passage after passage (Fig. 1). Since high mRNA expression of CBFA1, OPN, OPNp was found in low-passage, only cells in the 2nd passage were used in the experiments.

### Antibiotics preparation and exposure

Antibiotics in different concentrations were added to the cells in triplicate 24 h after seeding. Each drug was prepared in three different concentrations: 10 μg/mL, and 100 μg/mL and 1000 μg/mL (equal to 10 times and 100 times the clinical dosage, respectively); 10 μL of each of the antibiotic solutions was then added to the wells. Control groups were exposed to a saline solution with the same conditions without antibiotics (saline control). We used the same xCELLigence system and WST-1 assay as that in our previous tenocyte research [11] for cellular proliferation assessment. Cytotoxicity of the drugs on periosteal cells in comparison to that of the non-drug saline control was expressed as cell index adhesion curves for the xCELLigence system, and as optical densities in the WST-1 assay for the conventional culture plate method.

### Cell seeding into xCELLigence 96-well plates

Complete media (50 μL) was firstly put into each well of the xCELLigence 96-well plates. After equilibration to 37 °C, the plates were applied into the xCELLigence station; baseline impedance was then measured. All wells and connections were ensured to work properly within the acceptable limits. Under three different concentrations (5 × 10^3^ cells/cm^2^, 1 × 10^4^ cells/cm^2^, and 2 × 10^4^ cells/cm^2^), the cells were then seeded into the wells for a pilot test to select an optimal cell seeding density for antibiotics exposure.

### Software and data plotting of xCELLigence system

Software version 1.2.1 of xCELLigence system was used in this experiment to have electronic documentation regarding the experimental details. The cell index indicates the amount of cellular adhesion in each well with cell index approaching zero indicating absence of living cells or in a suspension of dead cells. With cellular attachment onto the electrode, the measured signal was confirmed to correlate linearly with the cell amount throughout the experiment.

### Cell proliferation in conventional WST-1 assay

Periosteal cells were cultured inside conventional 24-well plates and awaited to adhere overnight. The cells were exposed to the same antibiotics concentrations used for the tests inside the xCELLigence system on the next day. The culture medium was then refreshed every third day until the day 7 after drug exposure when cell growth was analyzed using a WST-1 kit (Roche, Basel, Switzerland). By using the colorimetric WST-1 assay that was based on the cleavage of tetrazolium WST-1 into orange formazan by mitochondrial dehydrogenases in viable cells, the level of orange formazan increases when mitochondrial activity increases and can be quantified through an E LISA Reader (MWG-Biotech, Ebersberg, Germany) at 450 nm with a reference wavelength of 245 nm.

### Quantitative real-time PCR assay

With completion of the ultimate impedance measurement, ribonucleic acid (RNA) was isolated from the cell culture in the xCELLigence 96-well plates using the TRIzol reagent (Invitrogen, Carlsbad CA) as we described in previous publication [11]. The uQuant software was used to measure the RNA quantity and purity (A260/280). RNA was reverse-transcribed into cDNA using 1 μg of mRNA and a High Capacity Reverse Transcription kit (Invitrogen, Carlsbad, CA). With 10–100 ng of cDNA as a template, real-time PCR was performed using the StepOne Real-Time PCR System (Applied Biosystems, Foster City CA). The resulting cycle threshold (Ct) values were normalized and analyzed using the standard curve method. By using the TaqMan Gene Expression Assays, expression levels of core-binding factor alpha 1 (Cbfa1), osteopontin (OPN), and osteopontin promoter (OPNp) were obtained, relative to the levels of glyceraldehyde-3-phosphate dehydrogenase (GAPDH), which was the endogenous control (Table 1).

**Table 1:** Primers for reverse-transcription PCR (RT-PCR) to determine osteogenic gene expression

Gene	Primer sequence
GAPDH	Sense: 5′-GCCTGGTCACCAGGGCTGC-3′ Antisense: 5′-TGCTAAGCAGTTGGTGGTGCA-3′.
Cbfa1	Sense: 5′-CCGCACGACAACCGCACCAT-3′ Antisense: 5′-CGCTCCGGCCCACAAATCTC-3′
OPN	Sense: 5′-CCAAGTAAGTCCAACGAAAG-3′ Antisense: 5′-ATGTCTGCTCCTGTAGTGG-3’
OPNp	Sense: 5’-CAGAATGCTATGTCCTCAGA-3′ Antisense: 5′-CGTCCTCATCCTCATCAATA-3’

*GAPDH* glyceraldehyde-3-phosphate dehydrogenase

*Cbfa1* core-binding factor subunit alpha-1

*OPN* osteopontin

*OPNp* ONP promoter

### Statistical analysis

Each experiment was repeated in triplicate. Cell proliferation in response to different antibiotic concentrations was compared by the independent t test regarding the cell index results in the xCELLigence system and optical density results from the WST-1 assays in conventional cultures. Correlations between cell index results from the xCELLigence system and cell proliferation results (based on the optical densities) from the WST-1 assays were assessed by the Pearson correlation analysis. Pearson’s correlation coefficients (r) of 0.4 to 0.69 indicate moderate correlation, whereas values between 0.7 and 0.99 indicate a high correlation. All statistical analyses were performed with SPSS 21.0 for Windows (SPSS Inc.; Delaware, Ohio).

## Results

### Selection of optimal cell seeding density

**Fig. 2:**
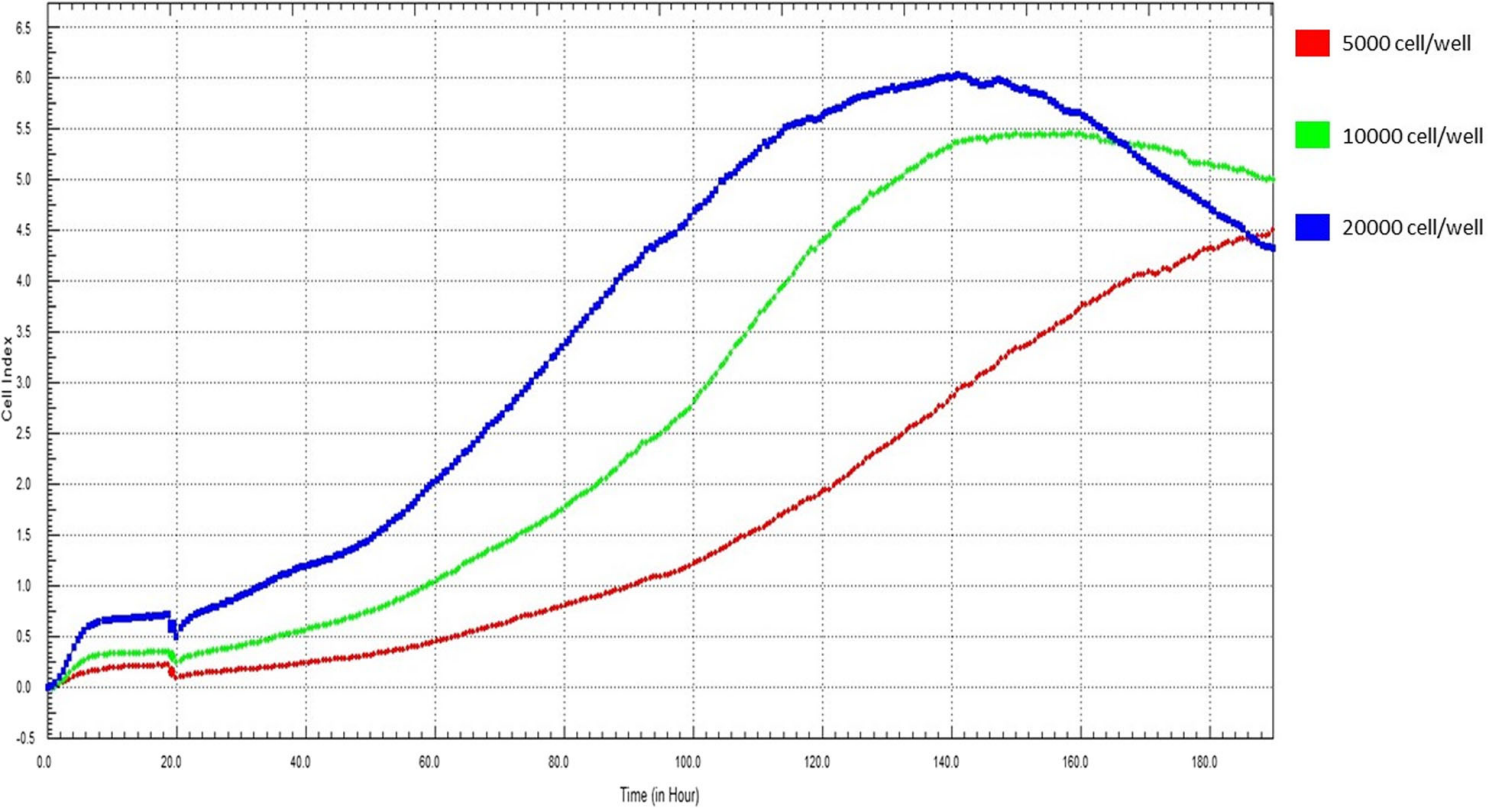
Determination of optimal cell number per well in xCELLigence 96-well plates

We used three different cell concentrations (5 × 10^3^ cells/cm^2^, 1 × 10^4^ cells/cm^2^, and 2 × 10^4^ cells/cm^2^) to determine the optimal number of cells for seeding in the pilot study (Fig. 2). In case of the rabbit periosteal cells, adhesion was slow initially. Then the cell index increased steadily over the first few hours after seeding, followed by a period of rapid proliferation. The proliferative phase was found at all seeding densities, and as expected, occurred more slowly at lower seeding densities. When 5 × 10^3^ cells/cm^2^ were seeded, the cell index was 0.4 ± 0.01 after 24 h of cell adherence and continuously increased to 1.4 ± 0.2 at the final time point (166 h). Cell indices for the seeding densities of 1 × 10^4^ cells/cm^2^ and 2 × 10^4^ cells/cm^2^ steadily increased and then slightly decreased, and changed from 0.6 ± 0.01 to 2 ± 0.2 and 1.2 ± 0.02 to 2.5 ± 0.12, respectively, at the final time point. After the pilot study, 5 × 10^3^ cells/cm^2^ was chosen as the optimal seeding density for the drug tests.

### Real-time cell index of periosteal cells exposed to antibiotics, and comparison with conventional cell proliferation assay

**Fig. 3:**
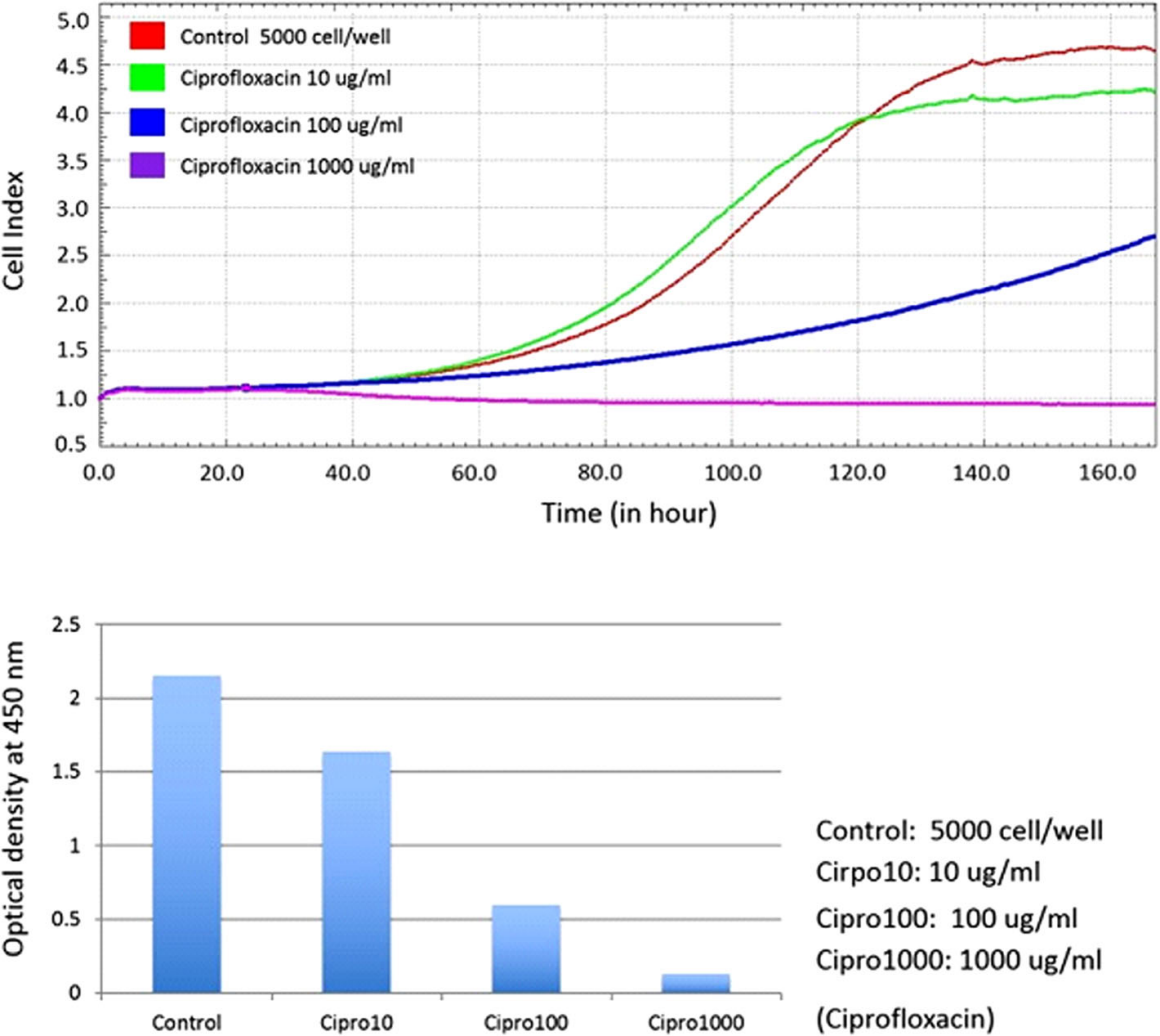
Ciprofloxacin cytotoxicity on periosteal cell proliferation. Ciprofloxacin cytotoxicity was expressed as cell index adhesion curves from the xCELLigence system (above) and as optical densities at 450 nm in the WST-1 assay from the conventional culture plate 7 days (below)

In the pilot study, a proper seeding density of 5 × 10^3^ cells/cm^2^ was selected for the xCELLigence system. After 24 h of cell adherence, 3 different concentrations of antibiotics were added to determine their real-time influence on cells. Cell proliferation was determined by the cell index in the xCELLigence system and WST-1 proliferation assays using conventional cultures (Fig. 3).

### Real-time cell index of periosteal cells exposed to antibiotics

**Fig. 4:**
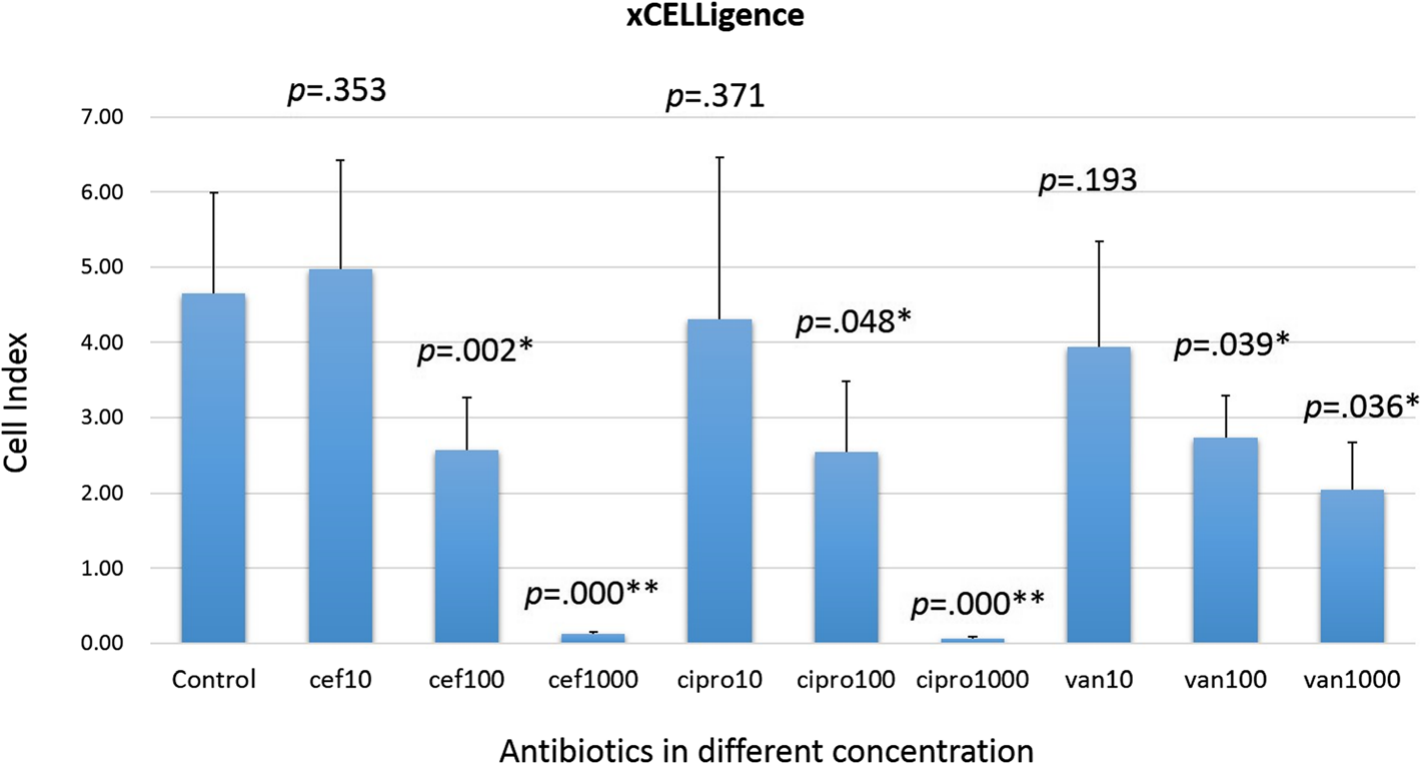
Cell index in the xCELLigence plates upon exposure to different antibiotics at day 7. Cef = cefazolin. Cipro = ciprofloxacin. Van = vancomycin. Unit of antibiotic concentration: μg/mL. The asterisk (*) indicates *p*-value less than 0.05, while double asterisks (**) indicate *p*-values less than 0.01

Cell index was measured at day 7 with a seeding density of 5 × 10^3^ cells/cm^2^ periosteal cells (Fig. 4). The average cell index was 4.66 ± 1.33 in the control group, and changed to 4.97 ± 1.45, 2.57 ± 0.7, and 0.12 ± 0.03 upon exposure to 10 μg/mL, 100 μg/mL, and 1000 μg/mL of cefazolin, respectively. The average cell indices were 4.31 ± 2.15, 2.54 ± 0.95, and 0.06 ± 0.03, upon exposure to 10 μg/mL, 100 μg/mL, and 1000 μg/mL of ciprofloxacin, respectively. The average cell indices were 3.94 ± 1.40, 2.73 ± 0.56, and 2.05 ± 0.62, upon exposure to 10 μg/mL, 100 μg/mL, and 1000 μg/mL of vancomycin, respectively. A significant difference was noted in case of all the antibiotics, when their dosages were increased to the medium and high concentrations (100 μg/mL and 1000 μg/mL) (Fig. 4).

### Conventional cell proliferation assay of periosteal cells exposed to antibiotics

**Fig. 5:**
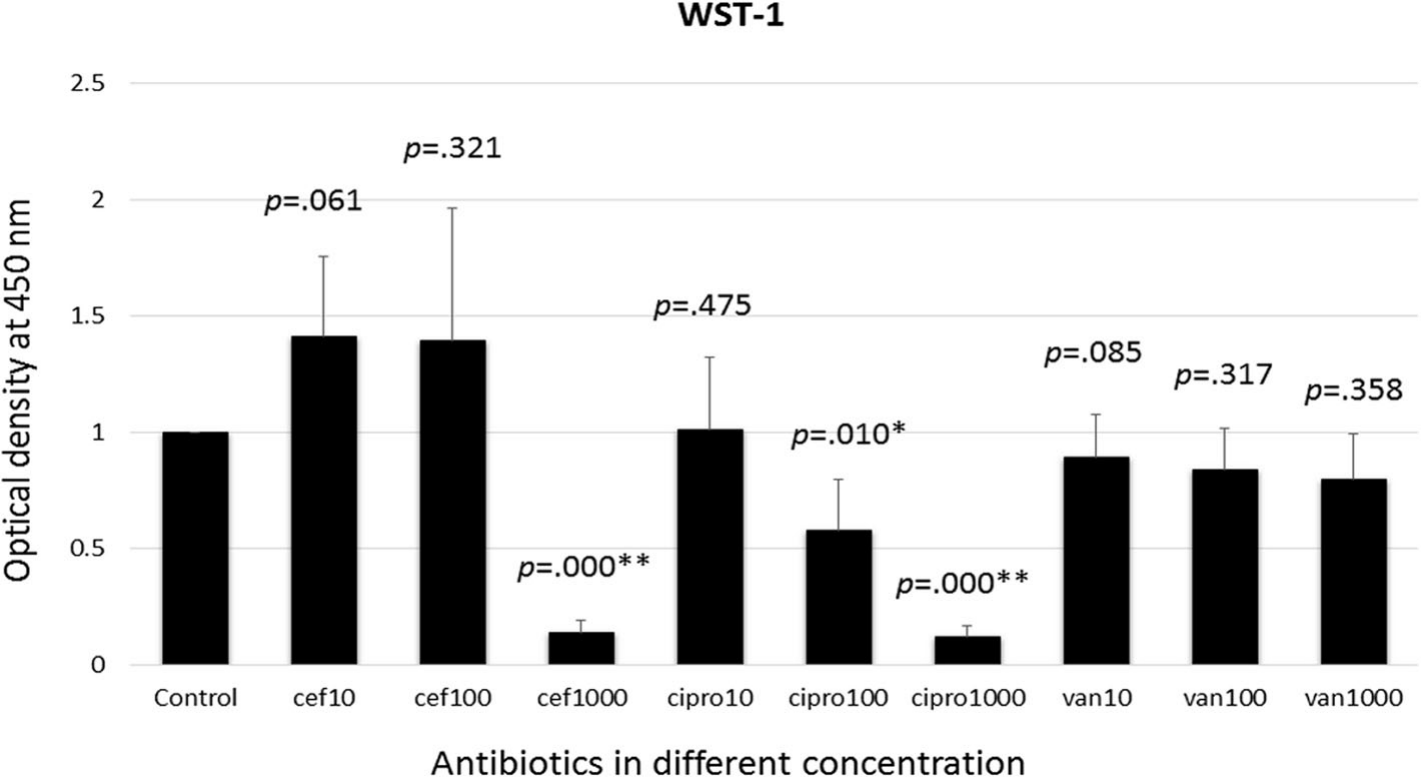
WST-1 assays at day 7 upon exposure to different antibiotics. Cef = cefazolin. Cipro = ciprofloxacin. Van = vancomycin. Unit of antibiotic concentration: μg/mL. The asterisk (*) indicates p-values less than 0.05, while double asterisks (**) indicate p-values less than 0.01

When the periosteal cells were exposed to different concentrations of antibiotics in the conventional culture system, at day 7, the WST-1 assays revealed a dose-related effect on cellular proliferation. The optical density averaged 1.41 ± 0.35 upon exposure to 10 μg/mL cefazolin, and then increased to 1.39 ± 0.57 and decreased to 0.14 ± 0.05, upon exposure to 100 μg/mL and 1000 μg/mL cefazolin, respectively. The optical density upon exposure to 10 μg/mL, 100 μg/mL, and 1000 μg/mL of ciprofloxacin averaged 1.01 ± 0.31, 0.58 ± 0.21, and 0.12 ± 0.04, respectively. The optical density upon exposure to 10 μg/mL, 100 μg/mL, and 1000 μg/mL of vancomycin averaged 0.89 ± 0.19, 0.84 ± 0.18, and 0.80 ± 0.19, respectively (Fig. 5). A significant dose-related difference was noted at a high dose (1000 μg/mL) of cefazolin, and medium-to-high dose of ciprofloxacin (100 μg/mL and 1000 μg/mL), but not for vancomycin.

### Correlation between the cell index from the xCELLigence system and WST-1 proliferation assays

**Fig. 6:**
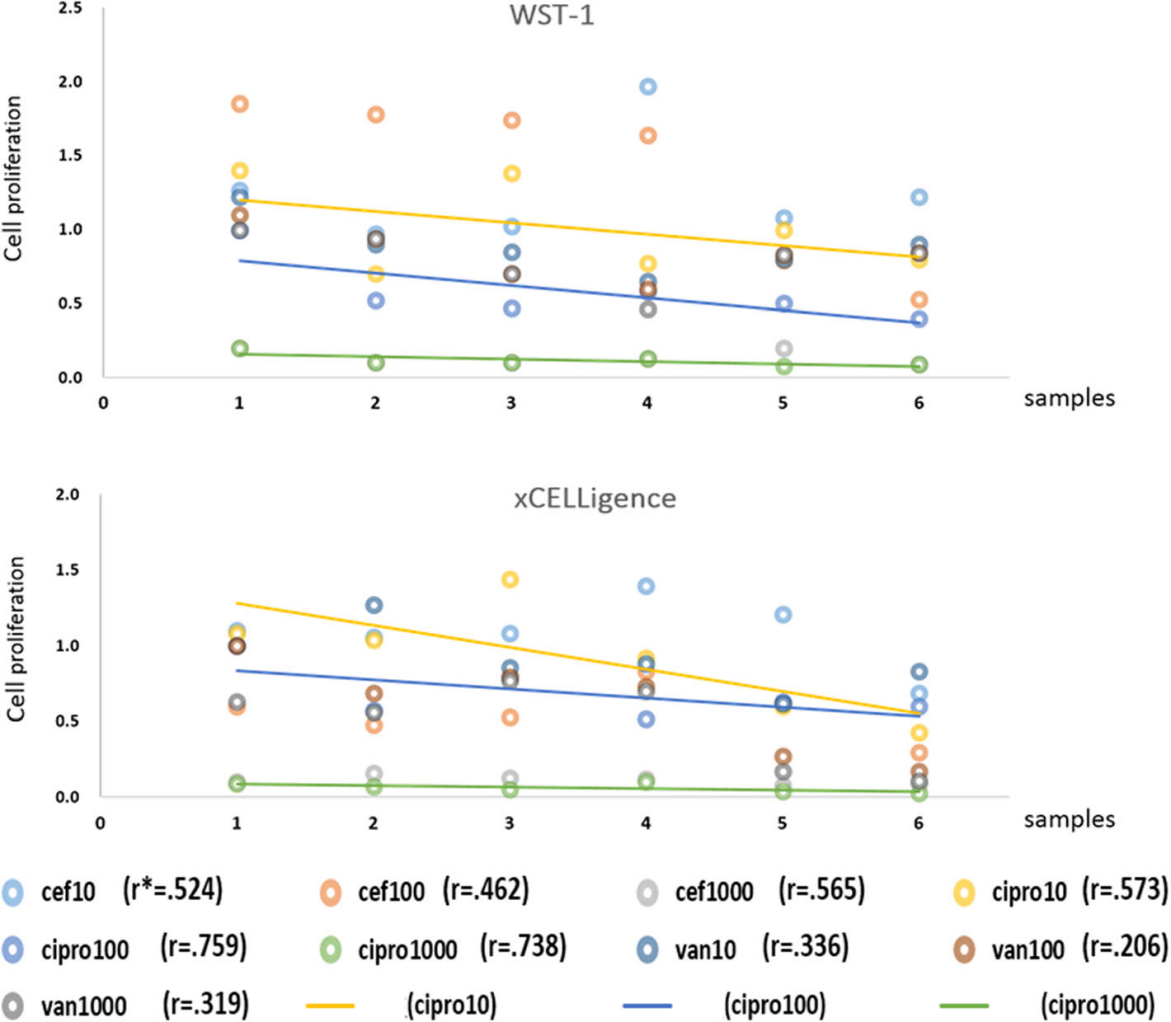
Correlations between the cell index and cell proliferation results. Correlations between the cell index results from the xCELLigence system (upper diagram) and cell proliferation results from WST-1 assays using conventional cultures (lower diagram) were assessed with Pearson’s correlation analysis. Pearson’s correlation coefficients (r) were calculated according to the concentrations of different antibiotics

The Pearson’s coefficient analysis revealed that the cell index ratio and WST-1 ratio for different antibiotic concentrations relative to the control groups showed a high correlation when the periosteal cells were cultured in moderate-to-high concentrations (100 μg/mL and 1000 μg/mL) of ciprofloxacin and showed a moderate correlation for cefazolin and low dose of ciprofloxacin (10 μg/mL) (Fig. 6). Correlation for vancomycin was low.

### Effect of different antibiotics on osteogenic gene expression

**Fig. 7:**
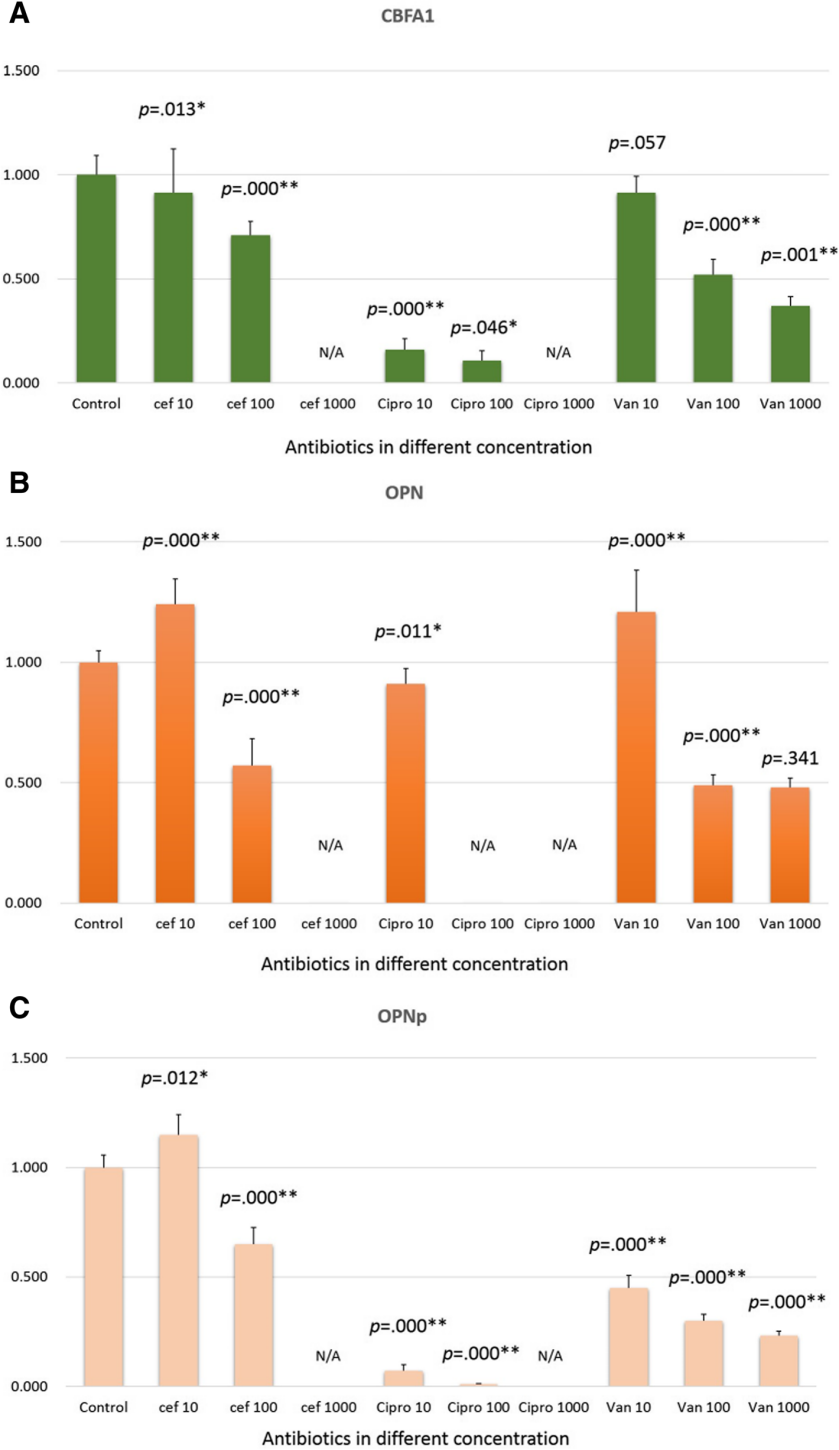
Osteogenic gene expression. The gene expression of osteogenic markers relative to the expression of GAPDH as the endogenous control upon culture in the xCELLigence system at 7 days. **a** cbfa-1. **b** OPN. **c** OPNp. Cef = cefazolin. Cipro = ciprofloxacin. Van = vancomycin. Unit of antibiotic concentration: μg/mL. The asterisk (*) indicates p-value less than 0.05, while double asterisks (**) indicate p-values less than 0.01

RNA was isolated from the cells cultured in the xCELLigence 96-well plates after final impedance measurement. To investigate the osteogenic activity of periosteal cells in the 96-well plates, mRNA expressions were obtained by real-time PCR for Cbfa1 and OPN, relative to GAPDH as the endogenous control (Fig. 7). A significant decrease in Cbfa1 expression was noted for low and medium doses of cefazolin and ciprofloxacin (10 μg/mL and 100 μg/mL); Cbfa1 expression was undetectable at high concentrations (1000 μg/mL). A significant dose-dependent inhibition of OPN expression was noted for low and medium doses of cefazolin (10 μg/mL and 100 μg/mL) and low dose of ciprofloxacin (10 μg/mL); OPN expression was undetectable with high doses of cefazolin (1000 μg/mL) and medium-to-high doses of ciprofloxacin (100 μg/mL and 1000 μg/mL). A significant dose-dependent inhibition of OPNp expression on exposure to cefazolin and ciprofloxacin was noted with low and medium concentrations (10 μg/mL and 100 μg/mL); OPNp expression was undetectable at high concentrations (1000 μg/mL). A dose-dependent inhibition of Cbfa1 expression was noted with the medium and high doses (100 μg/mL and 1000 μg/mL) of vancomycin; inhibition of OPN and OPNp expression was observed for all three concentrations of vancomycin.

## Discussion

Locally applied medications, including prolotherapy injections and antibiotic spacers, and pain management, are commonly used to treat musculoskeletal disorders [12, 13]. Local applications of antibiotics represent an important strategy for both the prevention and treatment of orthopedic infections; yet, only a small amount of prospective clinical data currently guides our practices. Additionally, concerns of systemic toxicity, as well as topical detrimental effects persist [14]. The periosteum is a well-vascularized osteogenic organ [15] and contains progenitor cells, which are responsible for callus formation and secondary bone healing after fracture, osteotomy, and even infections [16]. While local antibiotics are used to exert direct effects on bone at a cellular level [17], most studies have focused on their negative effects on stem cells and bone cells [15, 18, 19]. Since the periosteum is responsible for secondary fracture union whenever absolute stability and primary bone healing is unable to be achieved, basic research on dosage optimization needs to be performed and the antibiotic dose-related proliferative and osteogenic effects on periosteal cells need to be elucidated. Our study set up an automatic, cell-based impedance model to monitor the proliferation of periosteal cells under different antibiotic concentrations while current reports had demonstrated inhibitory effects of those three antibiotics in stem cells or bone-derived cells using traditional cellular assays [20–22]. An xCELLigence E-96-well plate was used, with an optimal seeding density of 5 × 10^3^ cells/cm^2^, to facilitate the continuous detection of cell proliferation and to avoid phenotype drift after prolonged maintenance in monolayer cell culture [23, 24].

Traditional cell studies using WST colorimetric assay [25] observed cell proliferation in conventional culture wells, which require large numbers of cells, large volumes of reagents, and are limited in their accessibility for high-resolution and time-lapse imaging. The microfluidic cell culture array is fabricated by soft-lithography technology and designed to maintain and monitor cell proliferation continuously [26]. With the integration of an electronic circuit into the microfluidic chip [27], this system allows the real-time detection of cellular response in a stable microenvironment in multiple assay conditions [28]. In our study, a dose-dependent inhibition on periosteal cell proliferation was observed, especially after treatment with medium-to-high antibiotic concentrations, in case of all three antibiotics. Similar dose-related changes were also observed at high antibiotic concentrations in case of the WST-1 assay using conventional cell cultures but not in medium concentration of vancomycin, which was not well correlated with the result detected by the xCELLigence system. Since vancomycin had been demonstrated to exhibit different inhibition activity in cellular osteogenesis [22], real-time monitoring of cellular proliferation with correlation to genetic expression is crucial in examining the drug toxicity in periosteal cells. Pearson’s coefficients showed a moderate-to-high correlation between the cell index from the microfluidic culture system and the optical densities from the WST-1 assay using conventional cell cultures; the impedance analyzer in the microfluidic culture system was more sensitive in detecting the dose-dependent inhibition of cell proliferation at medium antibiotic concentrations.

Real-time PCR analysis indicated that periosteal cells exposed to different antibiotics concentration in the xCELLigence E-96-well plate exhibited a dose-related decrease in osteogenic gene expression, which was compatible with the changes of proliferation index upon exposure to medium concentrations of the antibiotics. Although real-time PCR expressed a more sensitive detection in the dose-related changes, gene expression could be undetectable due to difficulty in the extraction of sufficient RNA in a small culture medium of 96-well plates upon exposure to high-dose antibiotics. This may highlight the distinctive potential of the xCELLigence system, which is a high-throughput analysis system, for clinical applications in identifying drug toxicity and predicting drug response in periosteal cells, and subsequent bone healing capability.

Our study has several limitations that warrant consideration. Firstly, neither this in vitro model, nor conventional cell cultures can represent in vivo conditions. Secondly, genotype survey of cbfa-1 and OPN may not fully and specifically represent the osteogenic expression of periosteal cells. Further animal studies using an infected nonunion model are mandatory to elucidate the influence of antibiotics on periosteal bone healing. Finally, only three dosages of antibiotics were selected for microfluidic assays; they may not adequately represent the integral characteristics affecting periosteal cell proliferation. In clinical applications, a more detailed survey using different antibiotic concentrations within the whole therapeutic range may be essential to clarify this dose-dependent relationship.

## Conclusion

Rabbit periosteal cells harvested from the tibial periosteum were successfully cultured at optimal seeding densities in xCELLigence 96-well plates; the microfluidic biosensor system allowed the dynamic monitoring of the cultured cells. Cefazolin and ciprofloxacin exhibited significant dose-related inhibition both in cellular proliferation and osteogenic expression. Such a non-invasive, label-free impedance-based analysis can serve as a feasible alternative to conventional WST-1 assays.

## Data Availability

The datasets generated during the current study are kept in the databank of Chang Gung Bone and Joint Research Center, and are available from the corresponding author on reasonable request.

## Abbreviations

Cbfa1: Core-binding factor alpha 1
DMEM: Dulbecco’s modified Eagle’s medium
FBS: fetal Bovine serum
GAPDH: Glyceraldehyde-3-phosphate dehydrogenase
IACUC: Institutional Animal Care and Use Committee
OPN: Osteopontin
OPNp: Osteopontin promoter
PBS: Dulbecco’s phosphate-buffered saline
PCR: Polymerase chain reaction
RNA: Ribonucleic acid
